# Recent Advances in Breast Cancer Research

**DOI:** 10.3390/ijms241511990

**Published:** 2023-07-26

**Authors:** Daniela Grimm

**Affiliations:** 1 Department of Biomedicine, Aarhus University, Ole Worms Allé 4, Aarhus C, 8000 Aarhus, Denmark; dgg@biomed.au.dk; Tel.: +45-21379702; Fax: +45-8612-8804; 2 Department of Microgravity and Translational Regenerative Medicine, University Clinic for Plastic, Aesthetic and Hand Surgery, Otto von Guericke University, Pfälzer Str. 2, 39106 Magdeburg, Germany; 3 Research Group “Magdeburger Arbeitsgemeinschaft für Forschung unter Raumfahrt- und Schwerelosigkeitsbedingungen” (MARS), Otto von Guericke University, 39106 Magdeburg, Germany

This Special Issue (SI), titled “Recent Advances in Breast Cancer Research”, covers 12 research articles and 1 communication in the field of breast cancer (BC) research. It includes publications reporting the results of cell biological, animal, and human studies.

BC is the second most common cancer in females, with skin cancer being the most prevalent cancer. BC accounts for about 30% (or 1 in 3) of new female cancers each year [1]. In recent years, BC incidence rates have increased by 0.5% per year [1]. The American Cancer Society provided estimates of BC in the United States for 2023, which are detailed in the following data: new cases of invasive BC will affect 297,790 female patients, and about 43,700 female deaths will occur as a result of BC. An estimated 2.3 million new cases of BC are diagnosed globally each year [2,3], and 684,996 BC-related deaths occur worldwide per year [3].

BC exhibits seven molecular BC subtypes with varying characteristic morphologies, treatment strategies, and future outcomes [4,5]. Patient survival depends on the tumor’s size, the specific hormone receptor profile, and tumor progression at the time of diagnosis.

The main BC treatment strategies are surgery and radiation-based treatment. Typical BC therapies include chemotherapy, immunotherapy, hormone therapy, and drug-targeted treatment strategies [6]. However, several available drugs can cause adverse effects. Another problem is the development of drug resistance in BC patients. Therefore, intensive efforts to advance cancer research, together with unified interdisciplinary attempts to identify novel strategies and drug targets, are necessary [6].

This SI covers several human studies (patients and specimens derived from human tissues) that used modern molecular biological technologies in BC research [7,8,9,10,11,12]. One study investigated the samples of male BC patients, focusing on the tumor microenvironment and infiltration of immune cells [9].

In addition, this SI includes seven cell culture studies [13,14,15,16,17,18,19]. Moreover, several papers of this SI focused on potential biomarkers [7,11,17] that predict either disease progression or a therapeutic response.

In total, this SI comprises 12 research articles [7,8,9,10,11,13,14,15,16,17,18,19] and one communication [12]. These 13 excellent papers were published as detailed in Table 1.

Fischer et al. [7] investigated 47 patients with metastatic BC and reported that the expression levels of circulating miR-200 family members were significantly increased during disease progression, which was predictive of circulating tumor cell (CTC) status. Both elevated CTCs and increased circulation of miR-200 content in blood plasma were associated with reduced OS and PFS. These factors are promising biomarkers for optimizing the clinical management of metastatic BC [7]. Another study investigated tumor specimens from 10 triple-negative breast cancer (TNBC) patients [8]. This BC type exhibited profound intratumoral heterogeneity. Therefore, single biopsy specimens may show only a portion of genetic aberrations of the entire tumor [8]. Genetic aberrations are involved in various cancer-specific biological processes, like tumorigenicity, induction of cell signaling, senescence, angiogenesis, migration, and response to treatment [8]. The authors concluded that medications used on the basis of the molecular profile of diagnostic biopsies may fail to remove the tumor, thus resulting in tumor recurrence. Knowledge of the molecular mechanisms that drive intratumoral heterogeneity in TNBC supports the development of new therapeutic targets.

A further article reports the results of a retrospective histological analysis of 113 cases of male BC, focusing on sTILs and programmed cell death ligand-1 (PD-L1) expression [9]. The authors demonstrated that a subset of male BC patients harbors an immunological environment characterized by an increase in sTILs with PD-L1 expression. Male BC are not only ER related and endocrine dependent, but also frequently HER2 low. These patients may benefit from immune checkpoint inhibitor therapy. In addition, frequent HER2-low status provides new options for anti-HER2 therapy in male BC patients [9].

Sokolenko et al. [10] investigated 229 BC patients. Among these patients treated via neoadjuvant chemotherapy (NACT) were 25 BRCA1 carriers and 204 women without recurrent BRCA1 alterations. NACT often results in a pathologic complete response (pCR). The authors found a lack of visible tumor cells in the post-NACT tumor bed to be a reliable indicator of the complete elimination of transformed clones [10].

Zaib et al. [11] investigated histological specimens of 97 patients with TNBC, showing that CD22 is highly expressed in this tumor type. The authors suggest that CD22 is a suitable prognostic biomarker in TNBC patients [11].

A further human study published as a communication focused on gene expression profiling of fibro-epithelial lesions (FELs) in the breast [12]. The authors studied the expression of 750 tumor-related genes in 34 FELs (5 fibroadenomas (FAs), 9 cellular FAs, 9 benign phyllodes tumors (PTs), 7 borderline PTs, and 4 malignant PTs). The overall gene expression profiles of benign PTs, cellular FAs, and FAs were similar. Borderline and benign PTs only slightly differed, whereas greater difference was detected between borderline and malignant PTs. This gene-expression-profiling-based approach supports further stratification of FELs, thus improving understanding of pathogenesis and diagnosis of BC [12].

Moreover, this SI covers several in vitro studies that investigated different human cell types [13,14,15,16,17,18,19]. Two such publications used data sourced from animal experiments [15,19].

Two studies focused on BC cells exposed to simulated microgravity conditions using an RPM, which is a device designed to create conditions of weightlessness on Earth [13,14]. Known microgravity-induced changes in human cancer cells include alterations in the cytoskeleton and changes in the ECM, adhesion, migration, differentiation, proliferation, survival, and apoptosis [20]. Differential expression of various genes and protein production and secretion were reported in benign and malignant cells [21,22,23].

Wise et al. [13] analyzed the supernatants of MCF-7 BC cells in order to determine extracellular vesicles (EVs). The cells had been exposed to an RPM for 5 or 10 days. A clear rise in released vesicles following RPM exposure was measured at both time points. Moreover, changes in the distribution of subpopulations related to surface protein expression were reported. Studying BC cells under microgravity led to an improved in vitro model that focused on changes in small EVs. Cancer research in space will extend our knowledge of cell communication in the tumor microenvironment and contribute to finding new therapies for BC [13].

Another microgravity experiment studied MCF-7 and MDA-MB-231 BC cells for 14 days using an RPM [14]. Both cell types grew in one of two phenotype forms: (1) adherent two-dimensional monolayers or (2) three-dimensional multicellular spheroids (MCSs). *ERK1*, *AKT1*, *MAPK14*, *EGFR*, *CTNNA1*, *CTNNB1*, *ITGB1*, *COL4A5*, *ACTB*, and *TUBB* genes of MCSs were differentially regulated in both cell lines. Bioinformatic analyses revealed a positive association between the real metastatic microtumor environment and MCSs regarding EGF/MAP signaling, focal adhesion, cytoskeleton, and the ECM, depending on the BC type. This long-term investigation improved pre-existing knowledge of tumor spheroid formation and growth [14].

The third cell culture study applied different types of BC cells and reported distinct roles for the Grainyhead-like 2 (*GRHL2*) gene in luminal and basal BC [15]. *GRHL2* gene silencing performed via a mouse model revealed a decrease in primary tumor growth and reduced the number and size of lung colonies. Altogether, *GRHL2* influences growth and motility. It is negatively associated with patient survival and growth suppression.

Meligova et al. [16] conducted a pharmacological study that investigated MCF-7 BC cells and their responses to antiestrogens and retinoids [16]. The authors showed that ERβ1 is a marker of responsiveness; in contrast, ERβ2 was shown to be an indicator of MCF7 cells’ resistance to antiestrogens alone and in combination with all-trans retinoic acid (ATRA) [16]. The authors concluded that the five unique hub genes (*PPARG*, *HIPK2*, *ZFP36L1*, *HMGB2*, and *ALDH1A3*) create a gene expression signature that specified the therapeutic response of ERβ1-expressing and ERα-positive BC cells to 4-hydroxytamoxifen and ATRA therapy [16].

Moreover, a BC cell study showed that NUF2 promotes BC development and, thus, serves as a new tumor stem cell indicator [17]. The findings of this study were as follows: the overexpressed NUF2 upregulated the proliferation and tumor stemness ability of BC cell lines MCF-7 and Hs-578T [17].

Piasna-Słupecka et al. [18] found that the young shoots of red beet are a richer source of total polyphenols that have anti-carcinogenic properties and exhibit higher antioxidant activity. The polyphenolic profile of the juice from young shoots of beetroot and the apoptosis mechanisms induced by subjecting the juices to in vitro gastrointestinal digestion and absorption were studied [18]. The authors demonstrated the antiproliferative and apoptotic effects of the evaluated types of beetroot juice, in particular those made of young shoots or roots that were subjected to the process of digestion and absorption in an in vitro gastrointestinal tract model, against BC cells [18].

A combined cell culture and animal study (mice) investigated *CCL2*’s role in mediating stromal interactions [19]. THP-1-derived macrophages and mammary fibroblasts were co-cultured for 72 h, which induced an M2 phenotype and a rise in CCL2 gene expression. Mice that overexpressed *CCL2* in the mammary glands were analyzed for global gene expression via RNAseq, showing upregulation of cancer-associated gene pathways. The *CCL2*-overexpressing mice showed enhanced macrophage infiltration and tumorigenesis [19].

Taken together, these 13 publications demonstrate novel findings in the field of BC research. The authors investigated several genes and molecular pathways, increasing our understanding of BC to aid improved diagnosis and the development of novel therapies. Studies that applied modern molecular biological approaches and bioinformatic analyses were published in this SI.

I wish to thank all of the authors who contributed to this SI. I am convinced that application of modern molecular biological technologies, together with a personalized medicine, will enable prevention and diagnosis of and new therapies for BC. Currently, many investigations are applying OMICS technologies and bioinformatics to identify new proteins that may serve as new mortality-decreasing drug targets or detect novel biomarkers that aid improved diagnosis of BC.

## Figures and Tables

**Table 1:** Contributions to the Special Issue “Recent Advances in Breast Cancer Research”.

Author	Title	Topics and Results	Type	Reference
Fischer C. et al.	Circulating miR-200 families and CTCs in metastatic breast cancer before, during, and after a new line of systemic treatment	Clinical study: 47 patients Determination of the expression of miR-200a, miR-200b, miR-200c, miR-141, and miR-429 and circulating tumor cells (CTC) status (CTC-positive 5 CTC/7.5 mL) at baseline (BL), which occurred after the first completed cycle of a new line of systemic therapy (1C) and upon disease progression (PD); MiR-200a, miR-200b, and miR-141 expression were reduced at 1C compared to BL. Upon PD, all miR-200s were upregulated compared to 1C; At all timepoints, the levels of miR-200s were elevated in CTC-positive patients compared to CTC-negative patients; Furthermore, heightened miR-200 expression and positive CTC status were associated with poorer OS at BL and 1C; In metastatic BC patients, the number of circulating miR-200 family members decreased after one cycle of a new line of systemic therapy, though these members were elevated during PD, which was indicative of CTC status.	Research article	[7]
Xu Q. et al.	A case series-based exploration of multi-regional expression heterogeneity in triple-negative breast cancer patients	Clinical study: 10 TNBC patients; RNA sequencing analysis of 2–4 regions per tumor; Gene expression signatures were categorized into three types of variability: between-patient (108 genes); between patient, across region (183 genes); and within-patient, between-region (778 genes) variability; Extensive intertumoral heterogeneity and regional ITH in gene expression and image-derived features in TNBC were determined.	Research article	[8]
Wise P. et al.	Prolonged exposure to simulated microgravity changes release of small extracellular vesicle in breast cancer cells	Supernatants of MCF-7 BC cells, which were harvested after 5 or 10 days of simulated microgravity, were created via a random positioning machine (RPM); Substantial increase in released vesicles following RPM exposure at both time points were recorded; Altered distribution of subpopulations and their surface protein expression were recorded; Minimal changes were noted between the time points, thus pointing to early adaption.	Research article	[13]
Brcic I. et al.	Tumor microenvironment in male breast carcinoma with emphasis on tumor-infiltrating lymphocytes and PD-L1 expression	Retrospective study of 113 cases of male BC (MBC) surgically treated between 1988 and 2015; Determination of stromal tumor-infiltrating lymphocytes (sTILs) and the expression of programmed cell death ligand 1 (PD-L1) and pan-TRK in MBC occurred; A subset of MBC patients harbored an immunological environment characterized by increased sTILs with PD-L1 expression; Potential benefits were provided via immune checkpoint inhibitor therapy. Frequent HER2-low BC of participants may offer novel anti-HER2 treatment options.	Research Article	[9]
Sahana J. et al.	Long-term simulation of microgravity induces changes in gene expression in breast cancer cells	14-day RPM exposure of MCF-7 and MDA-mb-231 cells; Study of cytoskeletal and extracellular matrix (ECM) factors, focal adhesion, and transmembrane proteins involved in MAPK, PAM, and VEGF signaling; Positive associations were recorded between the real metastatic microtumor environment and spheroids with respect to the ECM, cytoskeleton, morphology, different cellular signaling pathway key proteins, and several other components.	Research Article	[14]
Sokolenko A. P. et al.	Discrimination between complete and non-complete pathologic responses to neoadjuvant therapy using ultrasensitive mutation analysis: a proof-of-concept study in BRCA1-driven breast cancer patients	Clinical study: 229 patients; Lack of visible tumor cells in the post-NACT (neoadjuvant chemotherapy) tumor bed is a reliable indicator of the complete elimination of transformed clones; Failure of ultrasensitive methods to identify patients with minimal residual disease among pathologic complete response (pCR) responders suggests that the result of NACT is a categorical, rather than continuous, variable, with some patients being destined to be cured, while other patients ultimately fail to experience tumor eradication.	Research Article	[10]
Zaib T. et al.	Expression of CD22 in triple-negative breast cancer: A novel prognostic biomarker and potential target for CAR Therapy	Histological specimen of 97 TNBC patients; Significant associations between the size of tumors in TNBC patients and CD22 expression; CD22 shows potential as a prognostic biomarker; No significant correlation between overall survival of TNBC patients and CD22 expression; CD22 shows potential as a Chimeric antigen receptor (CAR)-T cell-based target in TNB.	Research Article	[11]
Wang Z. et al.	GRHL2 Regulation of growth/motility balance in luminal versus basal breast cancer	BC cell culture and animal study; Elevated Grainyhead-like 2 (GRHL2) identified in all BC subtypes and inversely correlated with overall survival in basal-like BC patients; Mouse basal A orthotopic trans-plantation model with silenced *GRHL2* gene; Distinct roles for GRHL2 in luminal and basal BC with respect to growth and motility were identified; in agreement with its negative association with patient survival, growth suppression is the dominant response to GRHL2 loss.	Research Article	[15]
Meligova A. K. et al.	ERß1 sensitizes and ERß2 desensitizes ER-Positive breast cancer cells to the inhibitory effects of tamoxifen, fulvestrant, and their combination with all-trans retinoic acid	Clones of MCF7 cells constitutively expressed human ERß1 or ERß2; Effects of antiestrogens 4-hydroxytamoxifen (OHT) and fulvestrant and retinoids (ATRA); MCF7-ERß1 and MCF7-ERß2 cells were sensitized and desensitized, respectively, to the antiproliferative effect of the antiestrogens, ATRA, and their combination, as well as to the cytocidal effect of the combination of OHT and ATRA; ERß1: marker of responsiveness; and ERß2: marker of resistance of MCF7 cells to antiestrogens alone and in combination with ATRA.	Research Article	[16]
Deng Y. et al.	NUF2 promotes breast cancer development as a new tumor stem cell indicator	NUF2: key role in the development and progression of BC by affecting tumor stemness in MCF-7 and Hs-578T cells; As a stemness indicator, it can serve as a marker for BC diagnosis.	Research Article	[17]
Piasna-Słupecka E. et al.	Young shoots of red beet and the root at full maturity inhibit proliferation and induce apoptosis in breast cancer cell lines	Effects of juice from young shoots of beetroot compared to juice from root at full maturity on human BC and normal cells; Juice from young shoots (native and digested form) was a significantly stronger inhibitor of the proliferation of MCF-7 and MDA-MB-231 BC cells than the native and digested juice from red beetroot; Regardless of the juice type, a significantly greater antiproliferative effect was measured in estrogen-dependent MCF-7 than in MDA-MB-231 BC cells; All analyzed types of beetroot juice, as well as juice from young shoots and the root, were subjected to digestion and absorption, exerting antiproliferative and apoptotic effects on both BC cell types.	Research Article	[18]
Archer M. et al.	CCL2-mediated stromal interactions drive macrophage polarization to increase breast tumorigenesis	*CCL2* is implicated in increased mammographic density and early breast tumorigenesis; THP-1-derived macrophages and mammary fibroblasts were co-cultured for 72 h; M2 phenotype and upregulated *CCL2* and other genes associated with inflammation and ECM remodelling; Global gene expression analysis of *CCL2* overexpressing mice; *CCL2* upregulated cancer-associated gene pathways and downregulated fatty acid metabolism gene pathways; Interactions between macrophages and fibroblasts regulated by *CCL2* can promote an environment that may increase BC risk, leading to enhanced early tumorigenesis.	Research Article	[19]
Li X. et al.	Gene expression profiling of fibro-epithelial lesions of the breast	Expression of 750 tumor-related genes in a cohort of 34 fibro-epithelial lesions (FELs) (5 fibroadenomas (FAs), 9 cellular FAs, 9 benign phyllodes tumors (PTs), 7 borderline PTs, and 4 malignant PTs; Genes involved in matrix remodeling and metastasis, angiogenesis, hypoxia, metabolic stress, cell proliferation, and the PI3K-Akt pathway were highly expressed in malignant PTs and less noticeably expressed in borderline PTs, benign PTs, cellular FAs, and FAs; Overall gene expression profiles of benign PTs, cellular FAs, and FAs were similar; Slight difference between borderline and benign PTs, as well as a higher degree of difference between borderline and malignant PTs; Macrophage cell abundance scores and CCL5 were significantly higher in malignant PTs than in all other groups; A gene expression-profiling-based approach led to further stratification of FELs.	Communication	[12]

## Abbreviations

ACTB	Actin Beta
AE	Adverse effect
AKT1	RAC-alpha serine/threonine-protein kinase
ATRA	All-trans retinoic acid
BC	Breast cancer
BCC	Breast cancer cells
CTC	Circulating tumor cells
COL4A5	Collagen type IV alpha 5 chain
CTNNA1	Catenin alpha 1
CTNNB1	Catenin beta 1
D	Day
ECM	Extracellular matrix
EGFR	Epidermal growth factor receptor
ERα	Estrogen receptor-alpha
ERβ1	Estrogen receptor-beta1
ERK1	Extracellular signal-regulated kinase 1
EVs	Extracellular vesicles
FAs	Fibroadenomas
FELs	Fibro-epithelial lesions
GRHL2	Grainyhead-like 2
Her-2	Human epidermal growth factor receptor 2
ITGB1	Integrin-beta1
MAPK14	Mitogen-activated protein kinase 14; p38
MCSs	Multicellular spheroids
NACT	Neoadjuvant chemotherapy
OHT	4-hydroxytamoxifen
OS	Overall survival
PD	Progression of disease
PTs	Phyllodes tumors
PD-L1	Programmed cell death ligand-1
RPM	Random positioning machine
sTILs	Stromal tumor-infiltrating lymphocytes
TNBC	Triple-negative breast cancer
TUBB	Tubulin-beta
